# Anterior Chamber Migration of Intravitreal Dexamethasone Implant in an Eye with Scleral-fixated Intraocular Lens

**DOI:** 10.18502/jovr.v15i4.7798

**Published:** 2020-10-25

**Authors:** Neha Goel, Aanchal Mehta, Jyoti Batra, Reena Choudhry

**Affiliations:** Department of Ophthalmology, ICARE Eye Hospital and Postgraduate Institute, Noida, Uttar Pradesh, India

##  PRESENTATION

**Figure 1:**
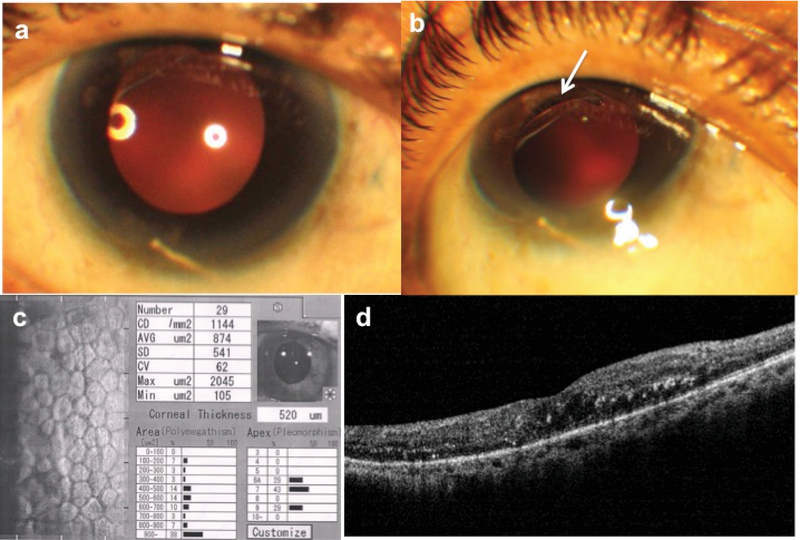
(a) Clinical photograph of the right eye showing a clear cornea, scleral-fixated posterior chamber intraocular lens (SFIOL) in situ, and an intravitreally administered dexamethasone (DEX) implant lying inferiorly in the anterior chamber. (b) A gap between the IOL edge and pupil margin could be seen in upgaze (white arrow). (c) Endothelial count of the right eye performed on the same day. (d) Spectral domain optical coherence tomography (SD-OCT) showing mild macular edema and hard exudates.

A 60-year-old woman with diabetes and hypertension presented with a decreased vision OD to 6/18 N10 due to cystoid macular edema (CME) following vitrectomy for removal of the dislocated posterior chamber intraocular lens (PCIOL) and scleral-fixated intraocular lens (SFIOL) implantation performed five months before. Spectral domain optical coherence tomography (SD-OCT) showed a central foveal thickness (CFT) of 484 µm. Intravitreal ranibizumab (LUCENTISⓇ; Genentech, Inc) was administered; however, the CFT increased to 533 µm after one month. She received intravitreal dexamethasone (DEX) implant (OzurdexⓇ, Allergan Inc.), which resulted in best corrected visual acuity (BCVA) improvement to 6/12 N10 and CFT reduction to 404 µm within a month. Three months later, CFT increased again to 677 µm and she received a second DEX implant. Seven weeks following the injection, she presented with pain, corneal haze, and intraocular pressure (IOP) of 38 mmHg. A week later, IOP decreased to 16 mmHg on brimonidine tartrate 0.2% and timolol maleate 0.5% BD; however, the DEX implant was seen in the anterior chamber (Figure 1a). Complete pupillary dilatation in this vitrectomized eye allowed the implant to migrate forward through the gap between the IOL edge and the pupil (Figure 1b).

There was no corneal edema and BCVA was maintained at 6/12 N10. Specular microscopy showed an endothelial count (EC) of 1144 cells/mm${}^{2}$ (Figure 1c). SD-OCT revealed a CFT of 365 μm (Figure 1d). A trial of wide pupillary dilatation with supine position failed to reposition the implant into the vitreous cavity. Pros and cons of implant removal were discussed and patient opted for weekly follow-up. Corneal clarity was maintained, EC was 1106, 1181, 1193, and 1205 cells/mm${}^{2}$ at each weekly visit, respectively, and IOP remained <20 mmHg without treatment. Within a month, the implant disappeared from the anterior chamber, although the CFT increased again to 711 µm. Due to the recent anterior migration of DEX implant, three monthly doses of intravitreal ranibizumab were administered. Suboptimal visual and anatomical results led us to inject DEX implant again, with care taken not to dilate the pupil fully at any time. She underwent four intravitreal DEX implants over the following year; BCVA remained stable at 6/12 N8 with a clear cornea, IOP < 20 mmHg in the absence of therapy, no recurrence of CME, and no anterior migration of the implant.

##  DISCUSSION

Intravitreal DEX implant is a safe and effective therapeutic option for post-surgical macular edema.^[1]^ Anterior chamber migration of the implant is a potential, though uncommon complication, in eyes with a compromised lens capsule, prior to vitrectomy and/or iris defects.^[2,3]^ Very few cases of migration in eyes with an SFIOL have been reported,^[2,4,5]^ as has occurred in this case, reinforcing that the presence of an IOL alone does not prevent the implant migration when the posterior capsule is not intact. As a precaution to minimize recurrent migration, the pupil size was kept reduced enough to cover the edge of the optic.

Corneal endothelial decompensation is the most serious complication resulting from the migration of implant into the anterior chamber.^[1,4]^ Early migration (within three weeks of injection) could result in higher incidence of corneal edema as higher rigidity of the implant in the first weeks could cause greater mechanical endothelial trauma.^[2]^ This would explain why our patient did not develop corneal edema and could avoid the additional procedure of immediate repositioning or removing the implant, which reduces the likelihood of permanent corneal edema.^[2,3]^

While vitrectomized eyes without an intact lens capsule are at risk for anterior migration of a DEX implant, this subset of patients may also benefit most from having a DEX implant administered for post-surgical macular edema. A regular post-injection follow-up can recognize and manage this rare, though potentially vision threatening, complication at the earliest. An individualized approach is recommended that takes into account the potential anterior segment complications as well as loss of drug effectiveness. A single episode of anterior chamber migration of the DEX implant should not be considered as a contraindication for further injections if warranted.

##  Financial Support and Sponsorship

Nil.

##  Conflicts of Interest 

There are no conflicts of interest.
